# Laparoscopic Repair of Iatrogenic Endoscopic Retrograde Cholangiopancreatography (ERCP)-Induced Duodenal Bulb Perforation in an Elderly Patient With Concomitant Type IV Hiatal Hernia: A Case Report

**DOI:** 10.7759/cureus.100990

**Published:** 2026-01-07

**Authors:** Manuel Damásio-Cotovio, Pedro R Conceição, Maria Miguel Evans, Maria Isabel Pereira, Madalena Siqueira, Maria Inês Matias, Martim Rente, Arnaldo Machado, Margarida Amaro, Manuel Carvalho

**Affiliations:** 1 Department of Surgery, Local Health Unit of Central Alentejo, Évora, PRT

**Keywords:** duodenal perforation, ercp, iatrogenic injury, laparoscopic repair, minimally invasive surgery, type iv hiatal hernia

## Abstract

Duodenal perforation is a rare but serious complication of endoscopic retrograde cholangiopancreatography (ERCP), particularly when anatomical distortions, such as large hiatal hernias, complicate endoscopic access. This case describes a 93-year-old patient with a type IV hiatal hernia who suffered an iatrogenic duodenal injury during a procedure for acute cholangitis. Due to the size of the defect and the patient's complex anatomy, an emergent laparoscopic repair was performed to reduce the hernia and repair the perforation. The patient's successful recovery highlights that prompt recognition and minimally invasive surgical intervention can be safe and effective, even in geriatric populations where anatomical challenges such as large hiatal hernias increase procedural risk.

## Introduction

Duodenal perforation is a rare and serious complication of endoscopic retrograde cholangiopancreatography (ERCP), with an incidence generally below 1% and significant associated morbidity and mortality. The pathogenesis is multifactorial, including direct trauma from the duodenoscope, sphincterotomy, and guidewire manipulation. Risk factors such as advanced age, altered anatomy, and procedural complexity increase susceptibility [1,2]. The American Gastroenterological Association (AGA) highlights that the thin duodenal wall and challenging anatomy contribute to the risk of these injuries [1].

Surgical repair remains the mainstay of treatment for large, intraperitoneal duodenal perforations (Stapfer type I) or when endoscopic closure is not feasible. Early recognition and prompt intervention are critical; delayed surgery after failed conservative management is associated with increased morbidity and prolonged hospital stays [2,4]. Surgical techniques typically involve primary closure of the defect; the addition of a T-tube duodenostomy is no longer recommended due to increased postoperative leak rates [2].

The presence of a hiatal hernia, while not a direct risk factor for perforation, complicates endoscopic access and increases procedural difficulty. In cases of large hiatal hernias, the stomach becomes redundant, and the gastroesophageal junction may be acutely angulated, causing the duodenoscope to loop and making pyloric access and stable positioning in the duodenum challenging. Furthermore, anatomical distortion can influence the surgical approach, necessitating careful intraoperative assessment [1,5].

## Case presentation

A 93-year-old autonomous male presented to the emergency department with a two-week history of epigastric pain, nausea, and choluria. Laboratory evaluation revealed significant hyperbilirubinemia (total bilirubin 6.32 mg/dL) and a marked cholestatic pattern, characterized by significantly elevated alkaline phosphatase (ALP 1202 U/L) and gamma-glutamyl transferase (GGT 2002 U/L). Additionally, there was evidence of a systemic inflammatory response, as evidenced by leukocytosis (WBC 11400/mm^3^) and an elevated C-reactive protein (CRP 8.0 mg/dL). A pre-admission computed tomography (CT) scan demonstrated choledocholithiasis and a large type IV hiatal hernia containing almost the entire stomach and a colonic loop. The patient was febrile (38.6ºC) but hemodynamically stable (blood pressure 142/61 mmHg). Following the initiation of empiric antibiotic therapy (piperacillin/tazobactam), he was admitted with a diagnosis of acute cholangitis.

**Figure 1:**
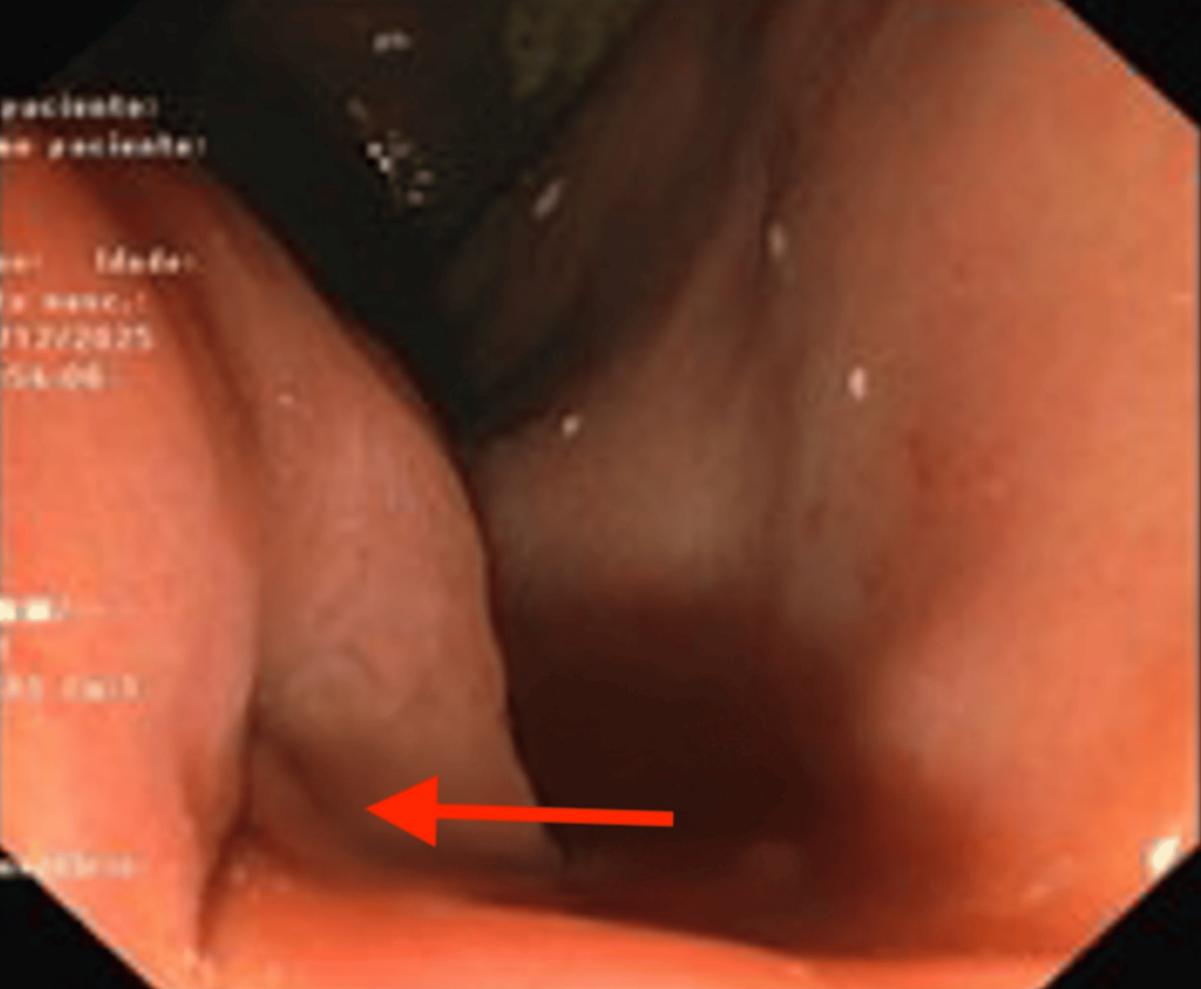
Endoscopic view of the gastric antrum and pylorus (arrow) showing anatomical distortion The red arrow indicates the pylorus.

**Figure 2:**
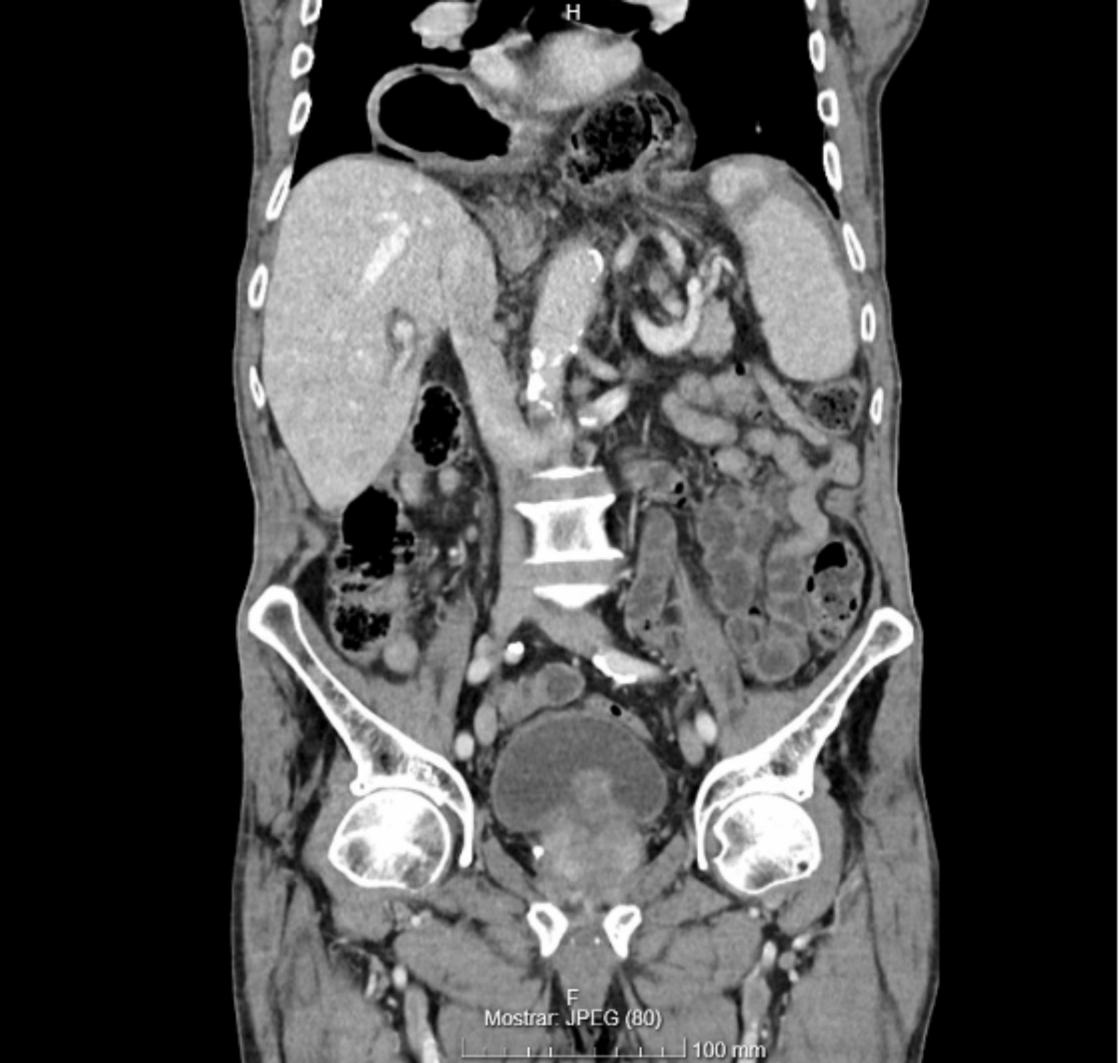
Coronal CT scan showing the large hiatal hernia

**Figure 3:**
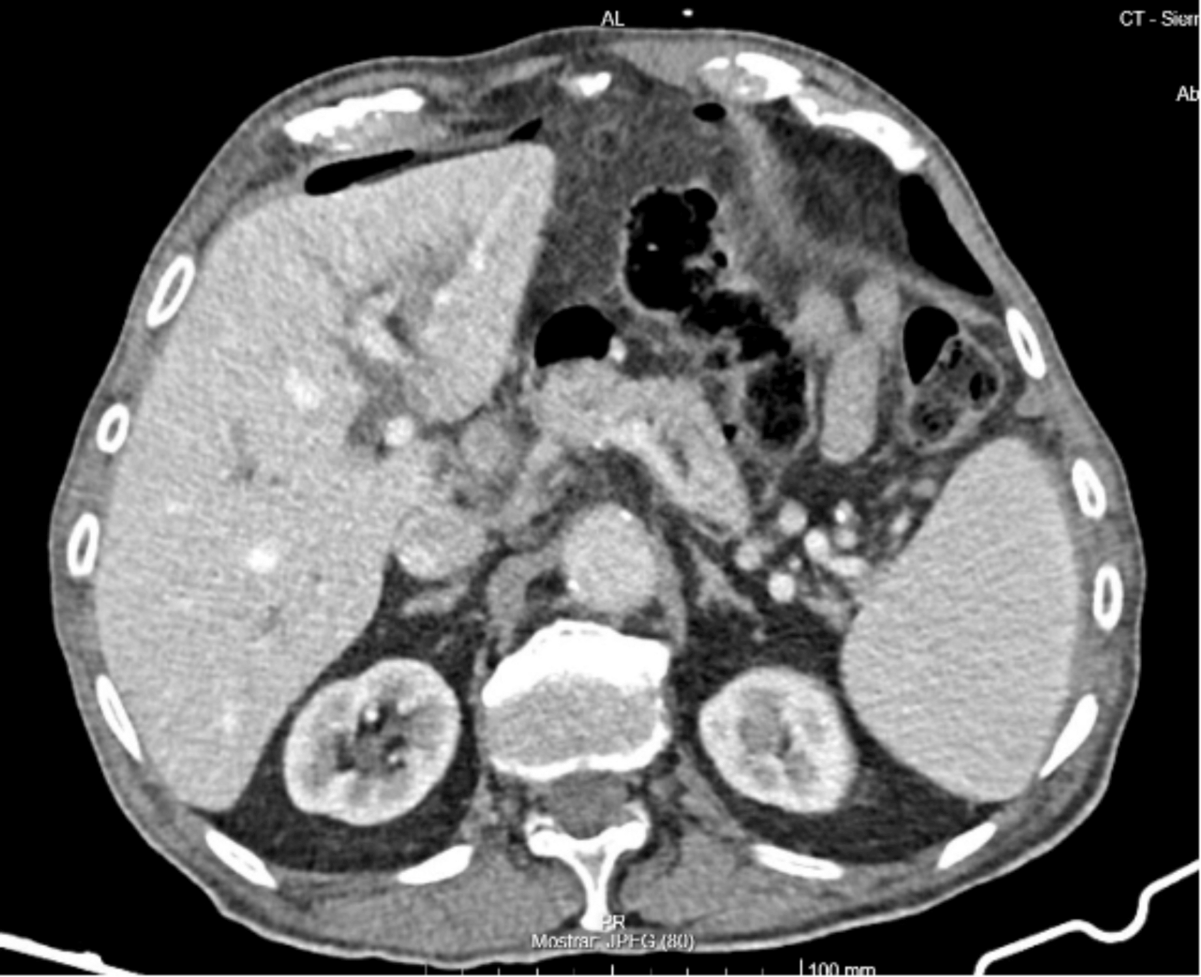
CT scan evidence of pneumoperitoneum

The patient demonstrated a clinical improvement, and an elective ERCP was performed. The endoscopist noted significant difficulty identifying the pylorus (Figure 1) due to anatomical distortion. Resistance was encountered past the pylorus, and a duodenal perforation was suspected upon visualization of extraluminal content. This procedure was immediately aborted before papillary cannulation or biliary drainage and without any further attempts at endoscopic closure, as it was considered that there were no conditions for a safe repair. A CT scan confirmed the hiatal hernia (Figure 2) and pneumoperitoneum (Figure 3). The patient was hemodynamically stable and consented to urgent surgery that was possible just six hours from diagnosis.

**Figure 4:**
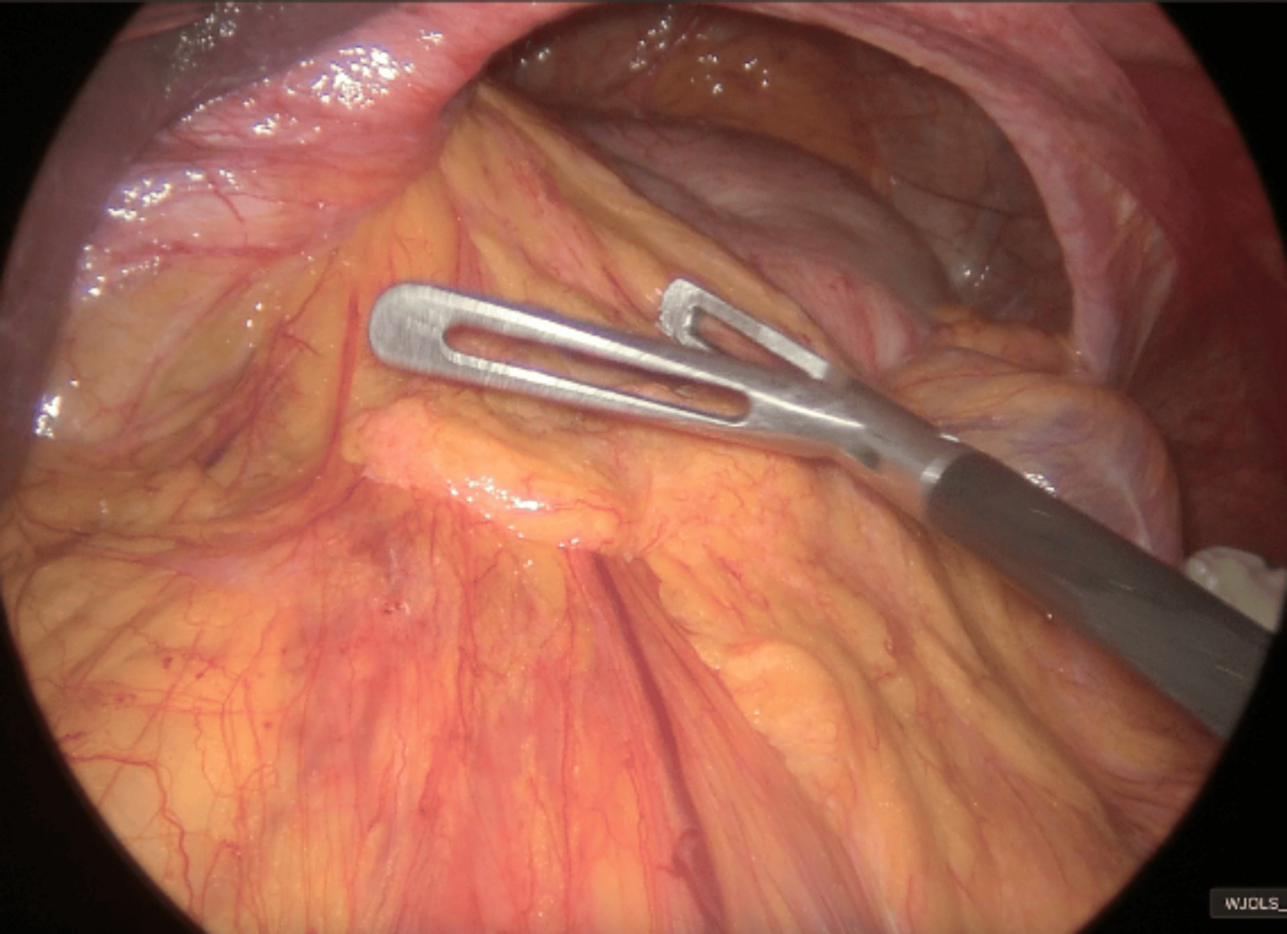
Intraoperative laparoscopic view of the type IV hiatal hernia reduction

**Figure 5:**
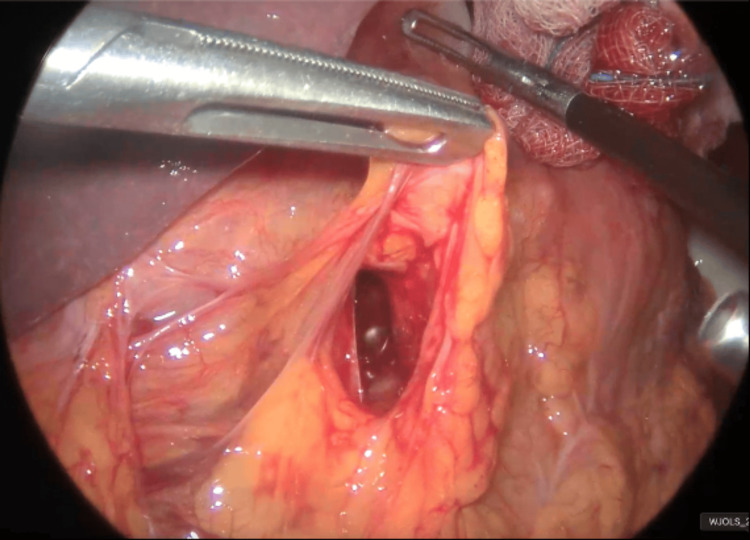
Opening of the lesser sac (omental bursa)

**Figure 6:**
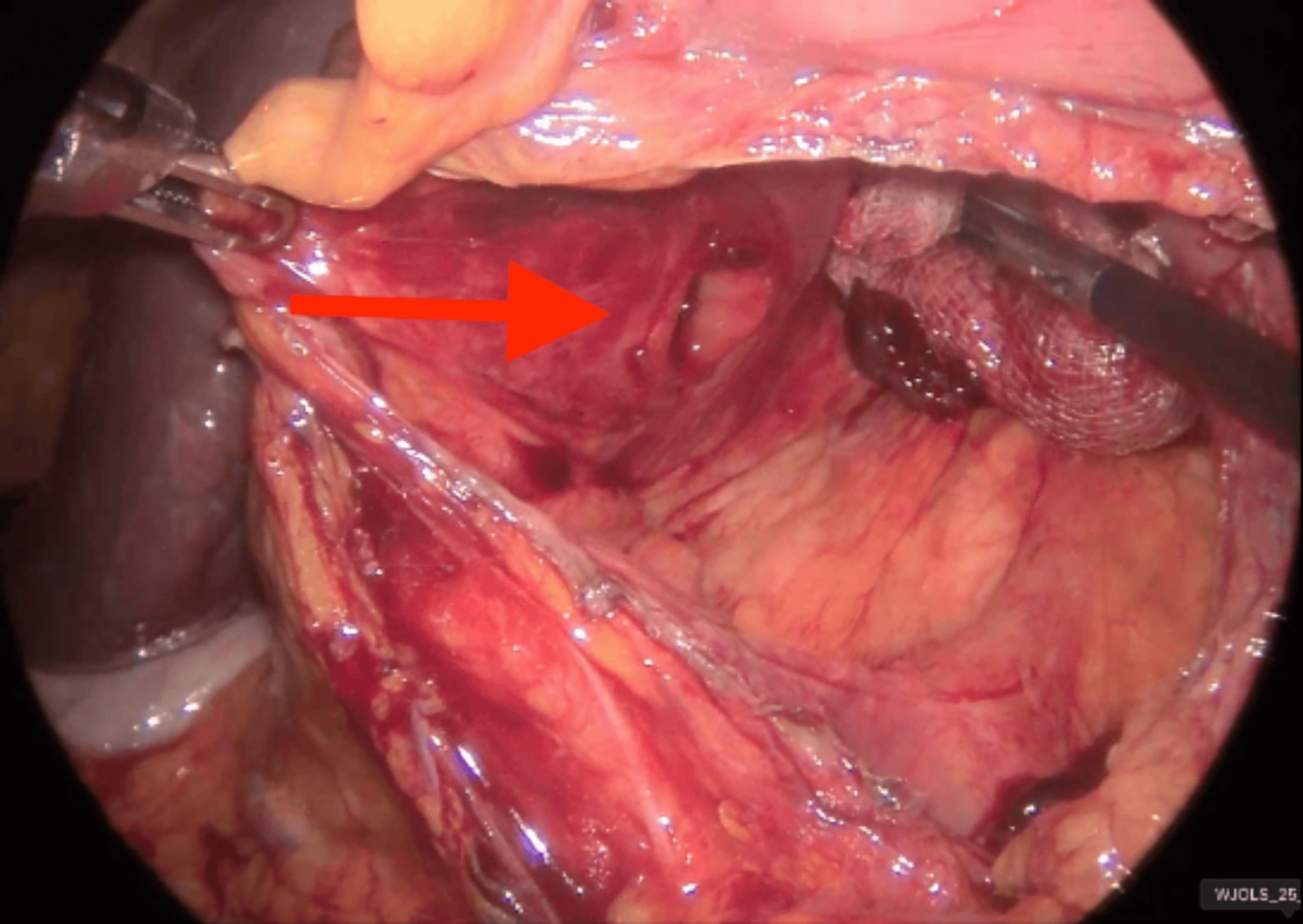
Visualization of the 2 cm perforation (red arrow) in the posterior wall of the duodenal bulb The red arrow indicates the perforation.

**Figure 7:**
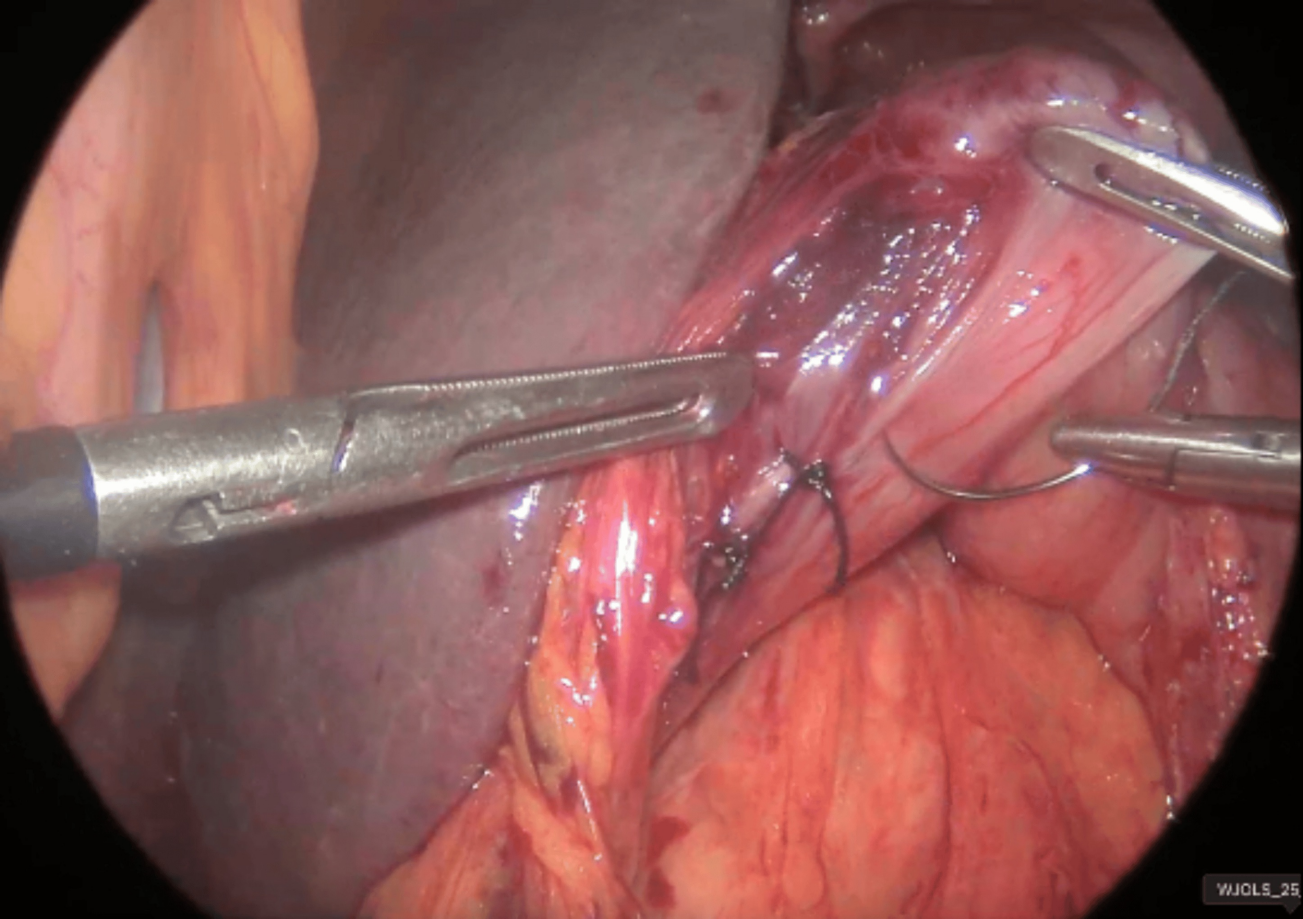
Laparoscopic view of the primary suture closure

A laparoscopic approach was utilized. A 12 mm trocar was placed at Palmer's point, followed by additional port placement. The hiatal hernia was reduced into the abdominal cavity (Figure 4). Upon entering the lesser sac (omental bursa), mucous and bilious-bloody content were noted (Figure 5). A 2 cm perforation (Stapfer type 1) was identified on the posterior wall of the duodenal bulb (Figure 6). The defect was repaired via primary closure using interrupted multifilament non-absorbable sutures (Figure 7). An intraoperative methylene blue test was negative. The goal of this urgent intervention was to avoid the development of a generalized peritonitis. For that reason, definitive solutions for the hiatal hernia or choledocholithiasis were not the priority and were not achieved in the same procedure.

The postoperative course was uneventful. The nasogastric tube, Foley catheter, and abdominal drain were removed, and the patient transitioned to a liquid diet on postoperative day two. He was discharged on the sixth postoperative day with normalized bilirubin and inflammatory and cholestatic markers, following a course of empirical therapy consisting of piperacillin/tazobactam and fluconazole, which also successfully managed the moderate acute cholangitis, although biliary drainage was not achieved.

## Discussion

Management of ERCP-related duodenal perforation in elderly patients with large hiatal hernias requires a multidisciplinary approach. A large hiatal hernia increases procedural difficulty and the risk of perforation due to impaired visualization, especially in older adults [1]. The American Society for Gastrointestinal Endoscopy (ASGE) recommends immediate fasting, IV fluids, and broad-spectrum antibiotics upon suspicion of perforation, followed by rapid assessment for surgical intervention based on clinical stability and imaging [6].

For large perforations similar to the one we report (Stapfer type I) or those with ongoing leakage and peritonitis, surgical repair is indicated. Endoscopic closure is often technically challenging in the setting of significant anatomical distortion [1,6]. Early surgical intervention (within 24 hours) is associated with improved outcomes [7-9]. In this case, prompt identification of the lesion and fast referral allowed for a repair within six hours from diagnosis. A low threshold for surgery is required in the geriatric population [6,10,11].

Laparoscopic repair has emerged as a viable alternative to open surgery, offering reduced postoperative pain and faster recovery [1]. In this case, the identification of the iatrogenic injury during the procedure was key to preventing diffuse peritonitis. Due to the anatomical complexity of type IV hernias and defect sizes >13 mm, endoscopic repair would be particularly difficult [6,12,13].

Given that the patient remained asymptomatic at the one-month follow-up and maintained a good performance status, the indication for further procedures was assessed. After weighing the risks and benefits, an elective cholecystectomy with common bile duct (CBD) exploration was planned, and the patient is currently awaiting surgery. A definitive correction of the hiatal hernia is not being considered because it was never symptomatic.

## Conclusions

Immediate laparoscopic repair of ERCP-related duodenal perforations is an effective and safe strategy in a stable patient when recognized early. The presence of a concomitant type IV hiatal hernia, while technically demanding, does not preclude a minimally invasive approach. This case underscores the importance of high clinical suspicion for iatrogenic injury in patients with distorted anatomy and suggests that advanced age should not be considered a contraindication to a successful laparoscopic surgical approach.

Despite the inherent advantages of laparoscopy, a definitive framework for selecting minimally invasive versus open repair in the context of iatrogenic ERCP duodenal perforations is not currently available. Developing a validated decision-making algorithm requires future studies to integrate patient-specific factors (age, comorbidities, performance status, and stability), injury-specific data (Stapfer type, size, time from diagnosis, and ERCP), and intraoperative variables (surgeon expertise, extent of peritoneal contamination). Establishing these benchmarks is essential for optimizing patient outcomes and standardizing surgical care.
